# Declines in mental health associated with air pollution and temperature variability in China

**DOI:** 10.1038/s41467-019-10196-y

**Published:** 2019-05-15

**Authors:** Tao Xue, Tong Zhu, Yixuan Zheng, Qiang Zhang

**Affiliations:** 1 BIC-ESAT and SKL-ESPC, College of Environmental Science and Engineering, Peking University, Beijing, 100871 China; 2 Ministry of Education Key Laboratory for Earth System Modeling, Department of Earth System Science, Tsinghua University, Beijing, 100084 China

**Keywords:** Environmental health, Environmental impact, Environmental health, Epidemiology

## Abstract

Mental disorders have been associated with various aspects of anthropogenic change to the environment, but the relative effects of different drivers are uncertain. Here we estimate associations between multiple environmental factors (air quality, residential greenness, mean temperature, and temperature variability) and self-assessed mental health scores for over 20,000 Chinese residents. Mental health scores were surveyed in 2010 and 2014, allowing us to link changes in mental health to the changes in environmental variables. Increases in air pollution and temperature variability are associated with higher probabilities of declined mental health. Mental health is statistically unrelated to mean temperature in this study, and the effect of greenness on mental health depends on model settings, suggesting a need for further study. Our findings suggest that the environmental policies to reduce emissions of air pollution or greenhouse gases can improve mental health of the public in China.

## Abstract (web summary)

Recent efforts to link mental health to environmental factors have focused on single predictors such as pollution or temperature anomalies. Here, the authors show that declines in self-assessed mental health scores were linked to increases in air pollution and temperature variability.

## Introduction

Mental disorders, the second leading contributor to the global disease burden, accounts for 7~13% of disability-adjusted life-years^1^. With improved medical services, many epidemiological studies have suggested an increasing trend toward longevity, but also a higher prevalence of morbidity and disability among the global population^2^. As mental illness has been ranked as the top risk factor for years lived with disability (YLD), accounting for 21~32% of the global YLD^1^, it is among the major driver of the global disease burden, which is transferring from mortality to disability/morbidity^2^.

A comprehensive understanding of relevant risk factors is required to mitigate mental disorders. The roles of conventional factors, such as drug abuse, maternal infection, perinatal depression, physical inactivity, hormonal changes, lifestyle, urbanization, and so on, have been well studied^3^. The epidemiological links between mental health and environmental factors are being increasingly examined in the context of the global challenges associated with climate change^4^. However, most extant studies have been performed in developed countries^4–11^. Indeed, there is limited evidence, particularly on a national scale, about such associations in developing countries, including China, where the adjusted prevalence of mental disorders has been reported as high as 17.5%^12^.

There are many psychological mechanisms that also make an epidemiological linkage between environmental factors and mental health biologically plausible. First, lack of greenness has been widely linked to mental disorders, including depression and anxiety in adults^5^, and cognitive dysfunction in children^6,7^. Many theories have been posited to explain these findings, including biogenics theory, the biodiversity hypothesis, restriction of physical activity, and social stressors^13^. Second, it has been shown that ambient pollutants, particularly fine particles, can cross the blood–brain barrier and thus damage the neurological system through introducing neuro-inflammation, neuronal signaling dysfunction, and immune responses^14^. Third, the mechanism underpinning the maintenance of body temperature suggests that mental health may be affected by ambient temperature. As some neurotransmitters, such as biogenic amines, play roles in both emotional and thermal regulation^15^, patients with mental disorders (e.g., schizophrenia) are prone to disturbances in thermoregulation^16^ and thus may find it difficult to maintain body temperature when exposed to highly fluctuating temperatures.

Although recent epidemiological studies have associated risk of mental disorders with individual environmental variables including high temperature^8,9^, poor air quality^17–20^, and lack of residential greenness^5–7^, questions about whether these associations are confounded by collinearity between factors remain unanswered. For instance, previous studies partially explained the link between mental health and residential greenness in terms of the superior air quality in greener places^21^. However, research that simultaneously incorporates multiple indicators is needed to identify the actual environmental risk factors. In addition, the health effects of long-term level of temperature have been well studied, whereas the potential risks of increased variability in the temperature to the health of the general public have to date only been suggested, i.e., by a recent epidemiological study^22^ that linked temperature variability with total mortality; however, these relationships have not yet been examined from the perspective of mental health.

This study used self-rated mental health scores (MHSs) from the China Family Panel Studies (CFPS)^23^ to make individual-level comparisons of the mental health of 21,543 adults from 25 populous provinces in China between 2010 and 2014 (Supplementary Fig. 1); we then linked these data to multiple environmental factors, including long-term level of temperature (*μ*_T_, annual mean of temperature), temperature variability (*σ*_T_, SD of daily temperature within a calendar year), air quality (measured by annual mean of fine particles with diameters < 2.5 μm [PM_2.5_]), and residential greenness (measured by annual mean of normalized difference vegetation index, NDVI). Specifically, the long-term exposures were evaluated in terms of the average annual values of the selected parameters within the county of residence of each individual (before the survey date), referring to previous studies on chronic environmental exposures^24^. This study, which used a difference-in-difference design^25^, is quasi-experimental in nature. As we compared each subject with her/himself, the study design, itself, controlled unmeasured confounders that varied inter-individually but not longitudinally. The difference-in-difference models directly regressed changes in MHSs with environmental variations, after multiple adjustments.

Statistical examinations of our data suggest that MHS decrease is robustly related to increase in PM_2.5_ or *σ*_T_, weakly related to NDVI decrease, and unrelated to *μ*_T_, among Chinese adults. According to the findings, the efforts to mitigate climate change and air pollution can bring extra benefits in aspect of human mental health.

## Results

### Summary statistics

This study involved 9474 (44.0%) urban adults and 12,069 (56.0%) rural ones. We found that more adults (40.5%) reported poorer mental health than unchanged (23.0%) or improved (36.5%) mental health from 2010 to 2014 (Supplementary Table 1). Indeed, the statistics (Supplementary Table 2) indicate that the decreasing trend in mental health was correlated with the feeling of depressed (*Q*_1_), nervous (*Q*_2_), and upset (*Q*_3_). Consistent with the trend toward global warming, the average *μ*_T_ increased by 0.98 °C, whereas the *σ*_T_ decreased by 0.55 °C. Probably benefiting from the land-use management^26^, the indicator of residential greenness, NDVI ( ∈ [−1, 1]) increased by 0.03. Co-determined by meteorological changes and the reduction in anthropogenic emissions resulting from China’s Clean Air Act^27^, the major species of ambient pollutant, PM_2.5_, decreased by 0.66 μg m^−3^.

### Mean temperature

**Fig. 1:**
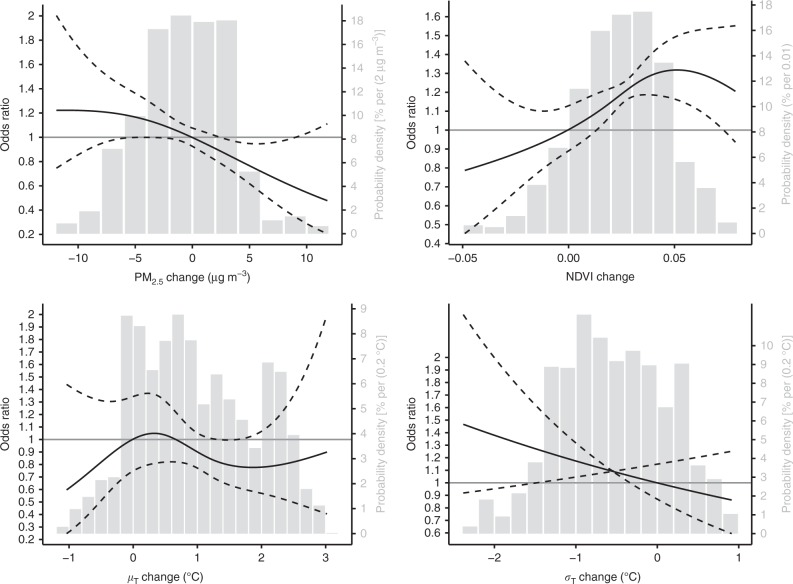
Exposure–response curves. The curves (the solid lines) with 95% confidence intervals (dashed lines) are estimated by the fully adjusted nonlinear effect models. The covariates include changes in alcohol consumption, education, migration, obesity, physical activity, and smoking status, as well as baseline age, alcohol consumption, education, diet type, gender, income, marital status, nationality, physical activity status, obesity status, area of residence, and smoking status in 2013. The histograms (gray bars) present the distributions of the environmental changes among the studied adults. For the exposure–response curves, please refer to the left *y*-axis; for the distributions, please refer to right *y*-axis

**Fig. 2:**
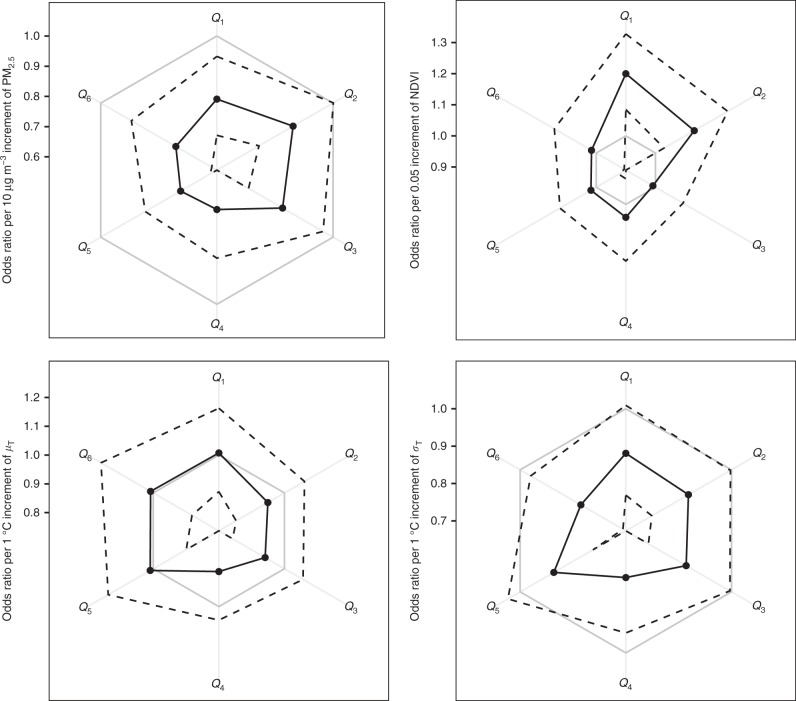
Environmental effects on different dimensions of mental health. The effects are evaluated by fully adjusted associations between the question-specific mental health scores and the four environmental factors. Black dots and black solid polygons: estimated odds ratios (ORs); black dashed polygons: corresponding 95% confidence intervals; gray polygons: references of no effect (OR = 1); gray radial lines: different dimensions of mental health; *Q*_1_: feeling depressed and incapability to cheer up no matter what you are doing; *Q*_2_: feeling nervous; *Q*_3_: feeling upset; *Q*_4_: feeling hopeless about the future; *Q*_5_: feeling that everything is difficult; *Q*_6_: thinking life is meaningless. Along a gray radial line, its interaction with a polygon presents the corresponding estimate or no-effect reference, for the dimension of mental health

Our results revealed a weak and complex association between *μ*_T_ and mental health. The nonlinear effect model indicated that either increased *μ*_T_ or decreased *μ*_T_ was associated to MHS decrease (Fig. 1). However, the pointwise confidence intervals (CIs) suggested the association was not statistically significant, which was consistent with the results of linear models (Fig. 2 and Supplementary Table 3). According to the fully adjusted model (i.e., model 5 in Supplementary Table 3), a 1 °C increase in *μ*_T_ was associated with a 3% (−11%, 15%) extra risk of MHS decrease. Both subregion and subgroup analyses (Supplementary Fig. 2) suggested the homogeneity of the weak association.

### Temperature variability

We found a significant association between the *σ*_T_ increment and MHS reduction, which remained robust after various adjustments (Supplementary Table 3) or model settings (Supplementary Table 4). The data showed that a 15% (3%, 25%) risk of MHS decrease was correlated with a 1 °C increase in *σ*_T_ (fully adjusted model; Supplementary Table 3). The nonlinear model further confirmed the negative association between changes in *σ*_T_ and changes in mental health status (Fig. 1). Based on the question-specific models, incremental changes in *σ*_T_ tended to be strongly linked to a higher probability of feeling nervous (*Q*_2_), upset (*Q*_3_), hopelessness (*Q*_4_), and meaninglessness (*Q*_6_) (Fig. 2). Although neither subregion nor subpopulation analyses revealed significant heterogeneity in the effect of *σ*_T_, this association may nonetheless vary slightly by geographical region (e.g., it may be weaker in Northern China than in Southeastern China) or inter-individually (e.g., it may be weaker in urban than in rural residents); these differences may be attributable to socioeconomic factors related to temperature maintenance facilities (e.g., owning an air conditioner). The results of double-exposure models indicated that the effect of *σ*_T_ was not considerably confounded by other environmental factors.

### Greenness

**Fig. 3:**
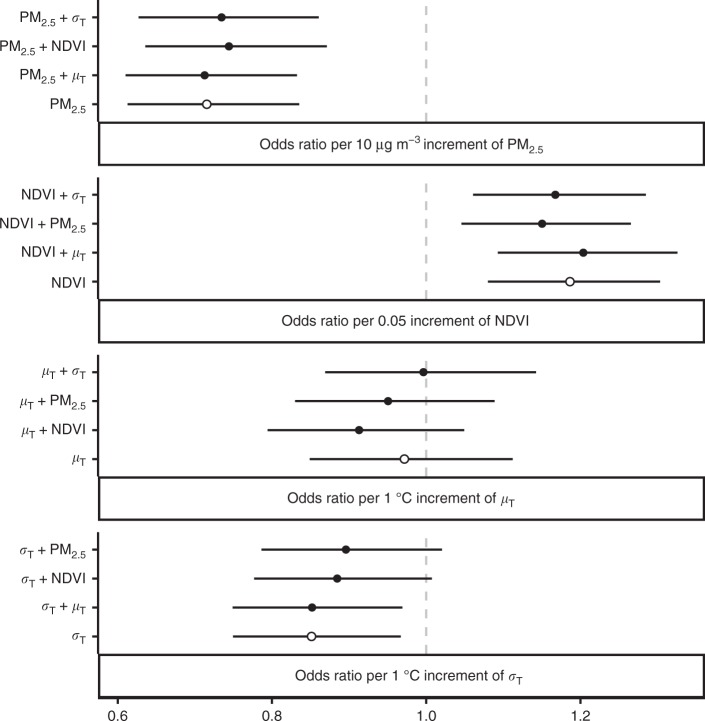
Results of the double-exposure models. In each panel, the fully adjusted odds ratios of an environmental factor with their 95% confidence intervals (black dots with error bars) estimated by the double-exposure models are compared with the estimate of the corresponding single-exposure model (black circles with error bars)

Our results are comparable to previous findings on the association between NDVI and mental health^5^. According to the fully adjusted models, every 0.05 decrease in the NDVI was associated to 19% (8%, 30%) risk of MHS decrease. Although this association was not considerably affected by adjustments for other environmental factors (Fig. 3), its significant level was sensitive to model settings, including adjusted covariates (Supplementary Table 3) and model assumptions (Supplementary Table 4). In addition, subgroup analyses suggested that some individual-level factors can modify the effect of the NDVI. Specifically, physical activity significantly enhanced this association (Supplementary Fig. 2), possibly because physically inactive adults may be relatively unaffected by the outdoor environment. Similarly, the question-specific results (Fig. 2) showed that increases in the NDVI may significantly alleviate feelings of depressed (*Q*_1_) and nervous (*Q*_2_).

### Air quality

Consistent with the existing evidences^10,11^, we found a significant association between higher levels of PM_2.5_ and MHS decrease. A 28% (16%, 39%) extra risk of reduction in MHS was associated with a 10 μg m^−3^ increase in PM_2.5_ (Supplementary Table 3, the fully adjusted model) and this effect remained robust after adjustment for different sets of covariates (Supplementary Table 3) and other environmental parameters (Fig. 3). Analogously, the association was not sensitive to different regression presumptions (Supplementary Table 4). Meanwhile, nonlinear analysis revealed a complex association for PM_2.5_ (Fig. 1). We found an effect threshold of ~ 5 μg m^−3^ for every increment in PM_2.5_ and the PM_2.5_ changes from 2010 to 2014 were above 5 μg m^−3^ for 8.5% of the study population. Subgroup analyses also reflected the complex effect of PM_2.5_ (Supplementary Fig. 2). For instance, our results suggest that PM_2.5_ had a significantly higher effect among the physically active adults. Potential heterogeneity in health effects of ambient particles has also been reported by previous studies^18^ and may be caused by variation in toxicity among different species of PM_2.5_, which may partially explain the apparent geographic variation in the effect (Supplementary Fig. 2).

## Discussion

In summary, according to our quasi-experimental population-level study on the effects of multiple environmental changes, declines in mental health of Chinese adults was strongly and robustly associated with increased *σ*_T_ or PM_2.5_, and plausibly related to decreased NDVI. Environmental changes have been evidenced as additional risk factors, which can impact on mental health, together with the well-studied factors, such as lifestyle and urbanization^3^. From 2010 to 2014, the overall trend of poorer mental health suggested that benefits from less variability of temperature and improved air quality did not offset the negative impacts from changes in other factors. For instance, the association between obesity and mental disorders is well known^28^, and there was an increased trend of obesity among our study population. The level of body mass index increased for 10.3% subjects, decreased for 5.6% subjects, and remained unchanged for the rest (Supplementary Table 1). However, the continuing efforts to mitigate environmental changes, such as clean air action^27^ and land-use management^26^ in China, is expected to improve mental health considerably. For instance, during 2013–2015, the national average of PM_2.5_ exposure was reported to decrease by 4.51 (3.12, 5.90) μg m^−3^ year^−1^, which was remarkable, compared with the PM_2.5_ reduction (0.66 μg m^−3^, Supplementary Table 1) in this study^29^.

The associations between mental health and environmental indicators in China have been explored. However, previous studies are based on data from local areas^14,17–20,30–32^ and their results have been mixed. For instance, a statistically significant association between hospital admissions for mental disorders and ambient exposure to PM_2.5_ was identified in Shijiazhuang^18^ but not in Shanghai^19^ or Beijing^20^. This divergence may derive from the heterogeneity of study populations, the use of different epidemiologic designs or statistical models, differences in the quality of the data, and so on. A national study, like this one, is needed to reevaluate the representative exposure–response curves among the general population. Taken together with these existing evidences, our findings confirm the epidemiological link between environmental changes and human mental health.

However, our findings are not conclusive because of the following limitations. First, mental health status was evaluated using a simple self-report questionnaire, which may call the quality of the data into question. The health outcome (MHS decrease or not) might be misclassified due to the potential errors in the questionnaire. Moreover, health outcome misclassification has been reported to bias the estimated association^33^. Analogously, misclassification may also have arisen from our approximation of long-term exposure levels according to annual and county-level averages. Any such exposure misclassification could lead to underestimation of the associations^33^. For instance, although the averaged exposure during the previous year might be representative to capture the environmental effects on mental health according to a sensitivity analysis (Supplementary Fig. 3), we might still ignore some risks from environmental changes in a longer term (e.g., lifelong exposure). Furthermore, although the difference-in-difference design could control unmeasured confounders, it has limited statistical power to detect weak associations, because variations in environmental changes (e.g., SD of ΔPM_2.5_: 4.0 μg m^−3^) were smaller than the overall spatiotemporal variations of those factors (e.g., SD of PM_2.5_ = 19.8 μg m^−3^). Finally, although this study examined and compared the psychological effects of four well-studied environmental factors, we may nonetheless have overlooked other associations between climate change and mental health.

Based on a nation-scale quasi-experimental study of Chinese adults, we derive representative exposure–response functions for indicators of air pollution and temperature variability, which can support the public health interventions for better mental health in China. Our results also reveal complexities underlying the epidemiological linkage between mental health and environmental changes, in the aspects of inter-individual susceptibilities, mutual confounders, and nonlinear curvatures, which should be explored by future studies.

## Methods

### Analytical diagram

The datasets utilized in our study are visualized in a diagram (Supplementary Fig. 4) with the data preparation procedures. Detailed steps in the diagram are illustrated in the following subsections.

### Study population

Our study population was drawn from the CFPS, an ongoing national survey on demographic and socioeconomic factors in China. The CFPS drew a representative sample of Chinese population using a multi-stage probability strategy with stratification, for multiple study purposes^23^. The CFPS surveyed > 30,000 adults and ~9,000 children from 25 provincial regions of China from 2010. Data on personal characteristics (e.g., age), socioeconomic status (e.g., education and income), behavior patterns (e.g., physical activity), lifestyle (e.g., diet type), mental health status, and so on were collected by trained interviewers using standard questionnaires. The study has been approved by the institutional review board at Peking University (Approval IRB00001052-14010). Although the CFPS collected the personal characteristics longitudinally, the surveyed variables slightly varied between years^23^. For instance, the surveys utilized the same mental health questionnaire in 2010 and 2014, but different ones in other years, which makes this study not qualified as a prospective cohort study.

In 2010, baseline mental health status was measured by a brief questionnaire based on the Center for Epidemiologic Studies Depression Scale test, consisting of six questions related to the following domains: feeling depressed and incapability to cheer up no matter what you are doing (*Q*_1_), feeling nervous (*Q*_2_), feeling upset (*Q*_3_), feeling hopeless about the future (*Q*_4_), feeling that everything is difficult (*Q*_5_), and thinking life is meaningless (*Q*_6_). Respondents were asked to rate the frequency with which they experienced these feelings on a scale ranging from 1 to 5 (1: almost every day, 2: 2–3 times a week, 3: 2–3 times a month, 4: once a month, and 5: never). Therefore, higher scores reflect better mental health. According to the CFPS user manual, the total score for the six questions constitutes an index of mental health status. In 2014, the mental health of subjects was examined using the same questionnaire. In total, 25,618 of the 33,600 adults surveyed in 2010 and 37,147 adults surveyed in 2014, participated in both evaluations. After excluding surveys with (1) incomplete answers to the mental health questionnaire or (2) a failure of geocoding (which will be described in following sections), the data obtained from 21,543 adults from 25 provinces (as shown in Fig. 1) during the first and second surveys were included in the final analysis. The characteristics of the involved samples were also compared with those of the total surveyed subjects in 2010 (Supplementary Fig. 5). The comparison showed that the data exclusion did not considerably changed the structures of the CFPS population, a representative sample of Chinese adults.

### Air quality

To examine the effects of the environmental factors that affect mental health, this study obtained data on air quality, residential greenness, and ambient temperature. To evaluate air quality, from a well-established product^29^, we obtained monthly maps of PM_2.5_ in China from 2000 to 2016, which had a spatial resolution of ~10 km × 10 km (in a regular grid of 0.1° × 0.1°). The gridded PM_2.5_ maps were estimated based on historical satellite measurements of aerosol optical depth and simulations of the Community Multiscale Air Quality Model based on historical emission inventories, using a machine learning model. The estimates have complete spatiotemporal coverage and were shown to be in good agreement with the independent in-situ PM_2.5_ values, based on the cross-validation (CV) results (*R*^2^ = 0.71; root mean square error [RMSE] = 17.8 μg m^−3^) on a monthly scale. In the CV, all observations of PM_2.5_ within a calendar year were used as the test data to validate the estimates from a model trained by the rest of the data and then the procedure was iterated (both retrospectively and prospectively) for all the PM_2.5_ observations during 2013–2016.

### Greenness

To assess residential greenness, we obtained a monthly product (MOD13A3, version 6) of the NDVI for China for 2009–2016, which had a spatial scale of 1 km × 1 km. As environmental exposures were evaluated at county level (as described below) due to the limited geographic information of the CFPS subjects, we did not obtain NDVI at a finer scale for computing efficiency. Satellite NDVI is a general index (varying from −1 to 1), which indicates the richness of green vegetation over the surface of the Earth; it has been widely used to measure long-term exposure to residential greenness. The NDVI data used in this study were also obtained from the moderate resolution imaging spectroradiometer (MODIS) products, which are freely distributed by the Application for Extracting and Exploring Analysis Ready Samples (EEARS): https://lpdaacsvc.cr.usgs.gov/appeears/ (accessed at May 2018).

### Temperature

To evaluate exposure to temperature, we obtained daily maps with a spatial resolution of ~10 km × 10 km (in a regular grid of 0.1° × 0.1°) from a data assimilation product for China from 2000 to 2016.

The surface temperature of the Earth can be obtained from multiple sources including routine climate monitors, satellite remote-sensing measurements, and climate model simulations such as the weather research forecast (WRF) model. Monitoring data are usually considered the gold standard, but is limited in spatial coverage, particularly in China. Although numerical outputs of climate models have a complete spatiotemporal coverage, they are less accurate. The temperature products of Earth-observing satellites, which scan the whole planetary surface within a 1–2 day time period, offer moderate coverage of spatiotemporal dimensions and have been utilized in health-related studies^34^. Recently, data assimilation products of monitoring and satellite-retrieved measurements have been derived to reduce errors in exposure assessment of ambient temperatures^35^. Inspired by such studies, we used the universal kriging^36^ approach to combine monitoring temperatures (*T*_m_), WRF-simulated temperatures (*T*_w_), and satellite temperatures (*T*_s_) to produce an optimal predictor of daily temperatures (*T*_optimal_) over China. Before universal kriging, we first prepared a product of satellite-based temperatures with complete spatiotemporal coverage (*T*_sc_ = [*T*_s_, *T*_s_^*^]), where missing values (*T*_s_^*^) of satellite measurements for each day were interpolated using the following equation: *T*_s_^*^ = *T*_w_^*^ + IDW(*T*_s_ − *T*_w_). In the equation, *T*_w_^*^ denotes the WRF output at the coordinates where satellite-retrieved temperatures do not exist and IDW(•) denotes an inverse-distance weighted average^36^ of the difference between the two measurements in the neighboring coordinates. Universal kriging is a two-stage model. In stage 1, we regressed *T*_m_ with *T*_sc_ and auxiliary variables, including satellite nightlight, altitude, and the monthly average of NDVI. In stage 2, we interpolated the residuals in stage 1 using kriging appraoch. The final estimates (*T*_optimal_) were obtained by adding the fitted values of regression in stage 1 and the interpolated residuals in stage 2. Due to computational complexities, universal kriging analyses were performed for each separate day. Therefore, empirical variograms were calculated and Matérn covariance function were fitted, by days.

In-situ observations of daily mean temperature (*T*_m_) during 2000–2016 were obtained from 225 monitors across China, from the global historical climatology network distributed by the National Centers for Environmental Information of the United States National Oceanic and Atmospheric Administration. Satellite-retrieved land surface temperatures (*T*_s_) were collected from level 3 (MOD11C1, version 6) MODIS products, which have a spatial resolution of 0.05° and generated valid data after 24 February 2000. Altitude data with a spatial resolution of 1 km were obtained from GTOPO30, which is a global digital elevation model developed from a US geological survey (https://lta.cr.usgs.gov/GTOPO30). Nightlight data in 2013 at a spatial resolution of 1 km were produced from the visible and infrared sensors of the Defense Meteorological Satellite Program (https://ngdc.noaa.gov/eog/dmsp.html). We also simulated daily maps of temperature (*T*_w_) during the study period using a well-developed WRF model (ver. 3.5.1) in China^37^.

The accuracy of the estimated temperature was evaluated using the tenfold CV approach, in which the in-situ observations were randomly divided and subjected to ten iterations of the validation procedure. According to the tenfold CV, the estimates were in excellent agreement with the daily in-situ observations (*R*^2^ = 0.96; RMSE = 2.46 °C), as shown in Supplementary Fig. 6.

### Exposure assessments

To protect their privacy, the detailed addresses of respondents were redacted from the open-access CFPS data. We obtained the six-digit administrative code (each code identifies a county-level geographic unit) for each subjects and then geocoded all CFPS samples into a map of county-level administrative boundaries in 2010, through matching their administrative codes. Finally, we identified 162 counties (Supplementary Fig. 1) that were consistent with those identified in the official reports of CFPS. In this county-level exposure assessments, we assumed that all residents of a county lived and commuted within the corresponding administrative boundaries. Therefore, the within-county variations in the long-term environmental exposures can be much smaller than the between-county variations. However, this presumption may not be valid for those residents who lived far from the county center. Considering that, we further validated the geographic information by comparing their reported distances from the provincial capital cities according to the community-level questionnaire with the calculated distances based on the geocoded county center. When the relative difference was >10%, all records from the community were excluded.

Due to the limitations of the geographic information, exposure levels were assessed at the county level. To evaluate long-term exposure to air pollution, we first averaged the gridded maps of PM_2.5_ into monthly county-level averages. All subjects from the same county were assigned to the same PM_2.5_ time series. Then, we calculated the annual average for each subject based on the PM_2.5_ value for the surveyed month and the values of the 11 preceding months (i.e., the 12-months moving average of PM_2.5_). When evaluating the long-term level of residential greenness, we processed the NDVI data in the same way as we processed the PM_2.5_ data, except that we calculated the population-density-weighted average instead of the direct average in each county. The 1 km × 1 km map of the population density in China was extracted from the 2010 Gridded Population of the World, which was also obtained from EEARS. The use of population-density weights reduces the misclassifications caused by the non-residential greenness of the NDVI, such as croplands and forests. Considering the complexity underlying the association between temperature and mental health, we prepared county-level time-series data on temperature in the same way as we prepared the PM_2.5_ data, but calculated the *μ*_T_ and *σ*_T_ during the year before the survey time to measure the long-term level of, and variability in, the temperature, respectively.

### Study design

We designed a difference-in-difference study to link changes in mental health to changes in long-term exposure to environmental factors. The difference-in-difference design has been widely used to explore the health effects of risk factors, such as ambient pollutants^25^, and is considered to yield results that are more relevant to causal relationships than those of cross-sectional studies. As the outcomes and exposure levels of a single subject are associated with each other in difference-in-difference studies, some confounders (e.g., genetic factors) that do not change with time are inherently controlled by the design.

In this study, we first derived the changes in the MHSs from 2010 to 2014, the long-term exposure levels to PM_2.5_, the NDVI, the *μ*_T_ and *σ*_T_, and the socioeconomic data (i.e., alcohol consumption, education, migration, obesity, physical activity, and smoking). Under the assumption that the various subgroups might show different mental health trends over time, we also used the baseline values of some socioeconomic variables (e.g., age, alcohol consumption, education, diet type, gender, income level, marital status, nationality, physical activity status, obesity status, area of residence (urban or rural), and smoking status) obtained in 2010 as additional covariates. To control the spatial autocorrelations in the outcomes, we first parameterized the coordinates of residential counties as a two-dimensional thin-plate spline function and further involved the term into the regression models. The optimal degrees of freedom for the spline term were automatically determined by the penalized method^38^. Such approach has been utilized in previous studies to examine the health effects of environmental factors and difference-in-difference analyses^25^.

### Statistical analyses

In purpose of good interpretability, the major analysis used a logistic model to examine the relationship between changes in total MHS and changes in each environmental variable after adjustment for multiple covariates, using the following equation:

1 $${\mathrm{Logit}}\left( {y_j} \right)\sim x_j\beta + {\mathbf{z}}_j{\mathbf{b}} + f\left( {{\mathbf{s}}_j} \right);\; \ldots \ldots$$

*y*_*j*_ = 1, for $Q_{i,\;j,\;2014} \ge Q_{i,\;j\;,\;2010}$,0, for $Q_{i,\;j,\;2014} < Q_{i,\;j,\;2010}$;or *y*_*j*_ *=* 1, for $\mathop {\sum}\nolimits_i {Q_{i,\;j,\;2014}} \ge \mathop {\sum}\nolimits_i {Q_{i,\;j,\;2010}}$,0, for $\mathop {\sum}\nolimits_i Q _{i,\;j,\;2014} < \mathop {\sum}\nolimits_i Q _{i,\;j,\;2010}$.

In the regression model, *i* or *j* denotes the index for mental health questionnaire or CFPS subject, respectively; *Q*_*i,j*_ denotes the score of the *i*^th^ question for the *j*^th^ subject; *y*_*j*_ denotes a binary variable to indicate the mental health change from 2010 to 2014; *x*_*j*_ denotes the corresponding change in an environmental factor (PM_2.5_, NDVI, *μ*_T_, or *σ*_T_); **z**_*j*_ denotes the individual-level covariates as described above; *f*(**s**_*j*_) denotes the spline function of spatial coordinates (**s**_*j*_); *β* and **b** denote the regression coefficients. Using the PM_2.5_ models as an example, the *x*_*j*_ was calculated as ΔPM_2.5, *j*_ = PM_2.5, *j*, 2014_ − PM_2.5, *j*, 2010_, where PM_2.5, *j*, *t*_ denotes the long-term exposure level for the *j*^th^ subject at the *t* year. The regression coefficient (*β*) for an environmental variable, *x*, can be interpreted as a logarithmic scale of odds ratios (ORs) for per-unit increments in *x*. An OR < 1 indicates that increment of *x* is associated to a lowered score (i.e., worse mental health).

Besides the models adjusted by different combinations of covariates (Supplementary Table 3), the major results also present (1) the nonlinear associations between mental health changes and environmental changes (Fig. 1), and (2) the double-exposure models (Fig. 3), based on modified versions of Eq. 1. To conduct the nonlinear analyses, we replaced the linear terms of the environmental variables with the thin-spline terms in the regression models. In addition, because the environmental variables were pairwise-correlated (Supplementary Table 2), they could act as confounders for each other. We used double-exposure models to explore these confounding effects. A double-exposure model simultaneously linked the health outcome with two environmental variables^39^. A comparison between a single-exposure model (e.g., a model of PM_2.5_) and the corresponding double-exposure model (e.g., a model of PM_2.5_ + NDVI) can reveal whether the estimated effect of the target variable (i.e., PM_2.5_) is sensitive to extra-adjustment of another variable (i.e., NDVI). A robust association suggests that the effect on mental health is more likely attributable to the target variable rather than its correlated variables.

In the sensitivity analyses, we first explored variations in the associations between total MHS and environmental factors using an indicator variable for three geographic regions and indicators for different demographic characteristics, including age, alcohol consumption, education, gender, income, obesity status, physical activity status, smoking status, and urban/rural residence (Supplementary Fig. 2). The variations were examined using interaction terms between the indicators and the environmental variables. Next, we examined alternative time windows for exposure to PM_2.5_ or *σ*_T_ (Supplementary Fig. 3), which had been estimated to be robustly linked with mental health in previous analyses. Finally, we modeled the MHS as alternative types of variable (Supplementary Table 4). In the major results, the changes in MHS were categorized into binary outcomes, to increase the interpretability of statistical analyses. However, the binned outcome might be insufficient to characterize the variations in mental health. Using modified version of Eq. 1, we also modeled the change in MHS as (1) a continuous outcome (Δ*Q* ∈ [−24, 24]) using a linear regression or (2) an ordinal outcome (Δ*Q* ∈ |−24, −23, …, 23, 24|) using an ordinal logistic regression (also known as the proportional odds model). Furthermore, we also directly associated MHS to environmental variables using the linear mixed-effect model, an alternative approach for the difference-in-difference design (Supplementary Table 4). The details of these alternative models are documented in Supplementary Table 4.

All statistical analyses were performed using R software (ver. 3.4.1; R Development Core Team, Vienna, Austria). The associations were presented by point-estimates with 95% CIs and their significances were evaluated by two-sided Wald’s tests.

### Reporting summary

Further information on research design is available in the Nature Research Reporting Summary linked to this article.

## Supplementary information

Supplementary Information

Reporting Summary

Description of Additional Supplementary Files

Supplementary Software

Source Data

## Data Availability

The population data (CFPS) that support the findings of this study are available from http://opendata.pku.edu.cn/. The NDVI data that support the findings of this study are available from https://lpdaacsvc.cr.usgs.gov/appeears/. The PM_2.5_ data that support the findings of this study are available from http://www.meicmodel.org/dataset-phd.html. The temperature data that support the findings of this study are available from https://www.ncdc.noaa.gov/ and https://search.earthdata.nasa.gov/.

## Code availability

R codes for the statistical models in the main-text are documented as the Supplementary Software. All R codes are available from the corresponding author upon request.
