# Author Correction: Declines in mental health associated with air pollution and temperature variability in China

**DOI:** 10.1038/s41467-019-11660-5

**Published:** 2019-08-06

**Authors:** Tao Xue, Tong Zhu, Yixuan Zheng, Qiang Zhang

**Affiliations:** 1 BIC-ESAT and SKL-ESPC, College of Environmental Science and Engineering, Peking University, Beijing, 100871 China; 2 Ministry of Education Key Laboratory for Earth System Modeling, Department of Earth System Science, Tsinghua University, Beijing, 100084 China

**Keywords:** Environmental health, Environmental impact, Environmental health, Epidemiology

Correction to: *Nature Communications* 10.1038/s41467-019-10196-y, published online 15 May 2019.

The original version of the Supplementary Information associated with this Article contained confidential geographical information in Supplementary Figs. 1 and 2. The HTML has been updated to include a corrected version of these figures. In corrected Supplementary Fig. 1, locations have been randomly placed within a 50 km radius of the surveyed sites and survey locations have been removed from Supplementary Fig. 2.

