# Supplementary material for: Declines in mental health associated with air pollution and temperature variability in China
Source: Nat Commun. 2019 May 15;10:2165. doi: 10.1038/s41467-019-10196-y (PMC6520357; doi:10.1038/s41467-019-10196-y)
Supplement: Supplementary file 1 — Supplementary Information [file 41467_2019_10196_MOESM1_ESM.pdf]

Supplementary Information

Title: Declines in mental health associated with air pollution and temperature variability in China

Authors: Xue *et al.*

Included Files:

Supplementary Figures 1-6

Supplementary Tables 1-4

Supplementary Software

Supplementary Figures

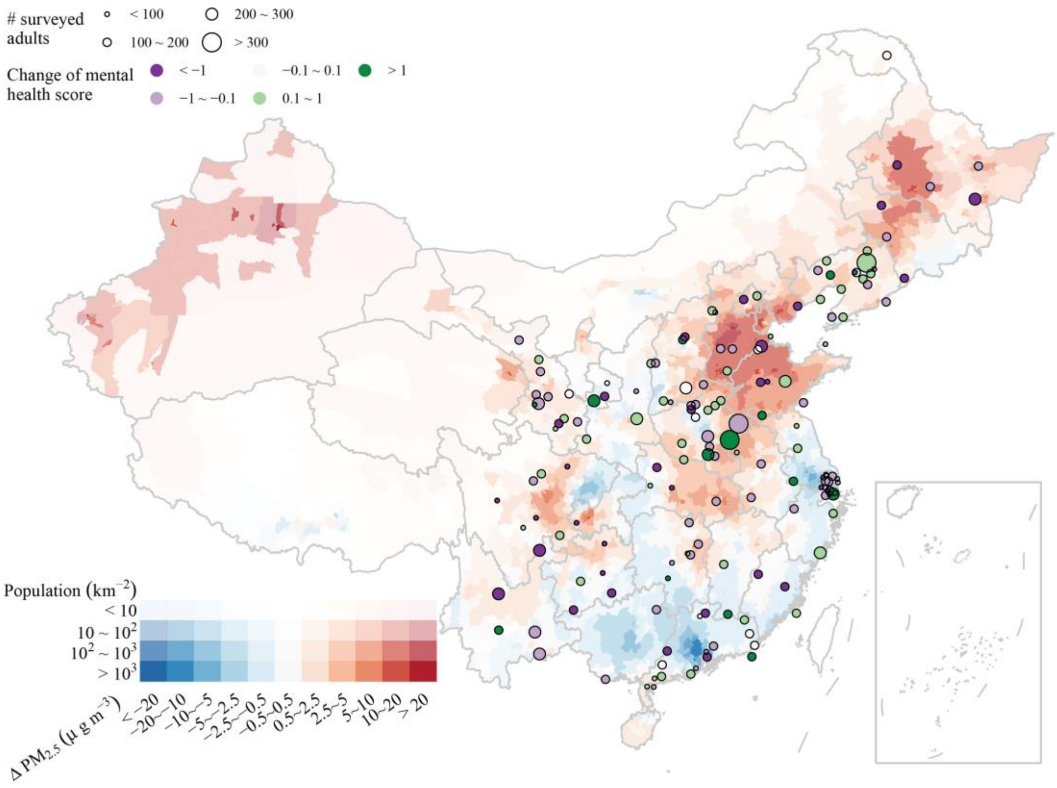

Supplementary Figure 1 Locations of surveyed counties and changes in mental health and related long-term exposure to $PM_{2.5}$ from 2010 to 2014. To ensure that respondent confidentiality is maintained, we randomly displaced the exact coordinates of the surveyed counties, and thus each displayed location has a random error of 50 km.

(a)

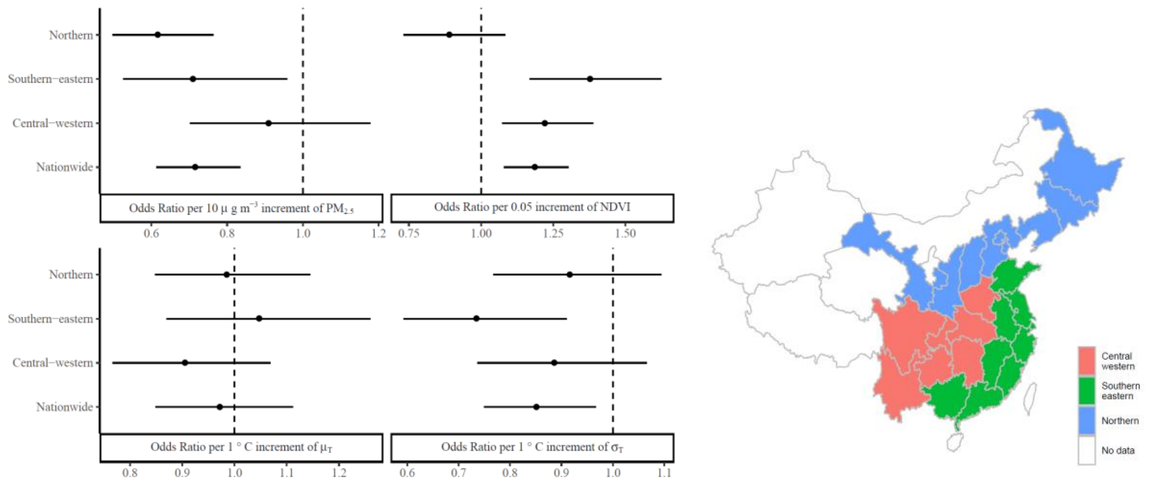

(b)

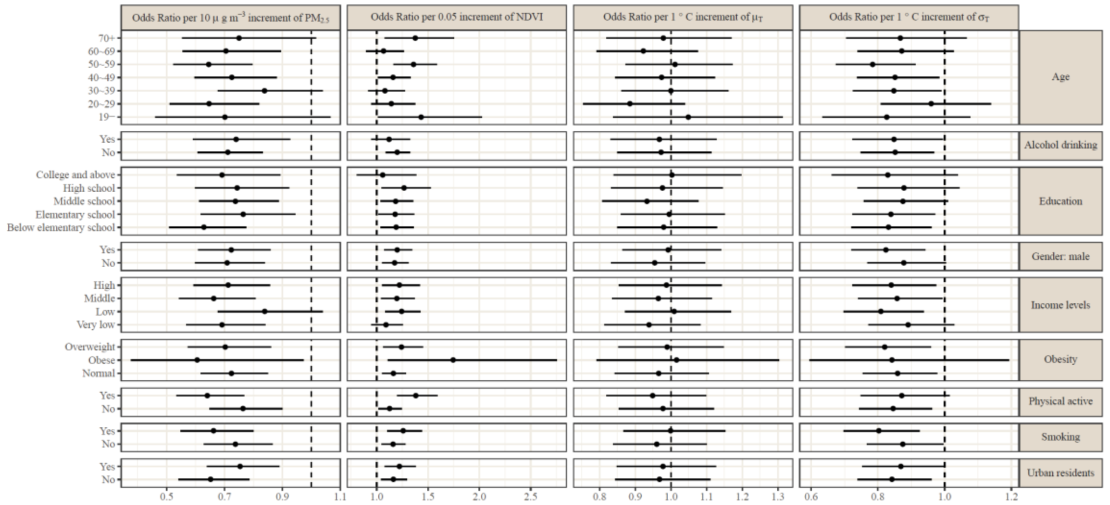

Supplementary Figure 2 Variations in the associations between mental health scores and environmental variables. Panel (a) presents the variations by geographic regions. Panel (b) presents the variations by demographic subgroups. The variations were examined by effect-modification analyses. Each panel presents the adjusted odds ratios with 95% confidence intervals (black dots with error bars) for one modifier. Effect-modification results showed that rural residents were significantly more sensitive than urban residents to the effect of fine particulate matter ($PM_{2.5}$), and that physical activity significantly strengthened the association between the normalized difference vegetation index (NDVI) and mental health. Other effect-modifications were statistically non-significant.

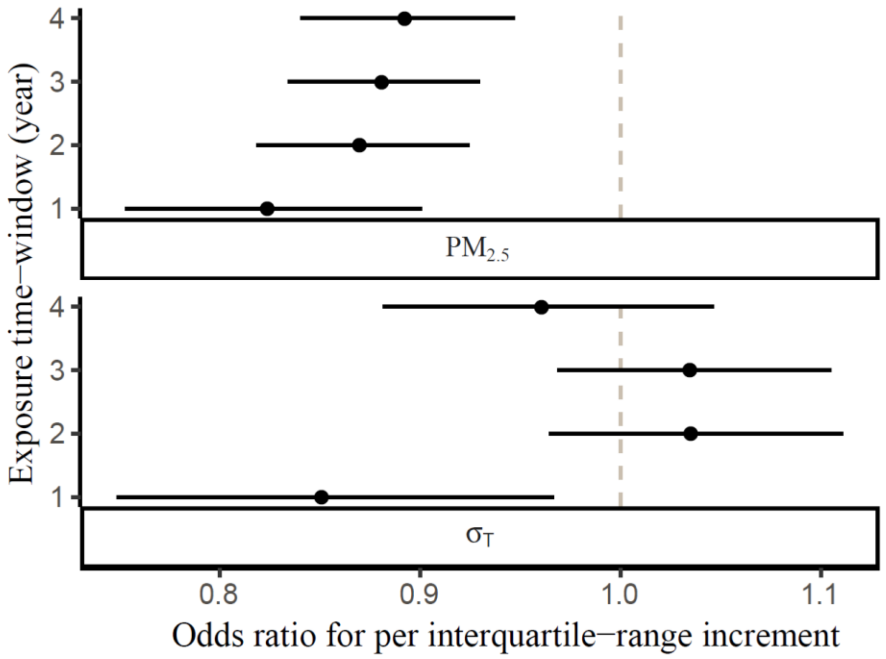

Supplementary Figure 3 A sensitivity analysis to explore the exposure time-window for $PM_{2.5}$ and temperature variability (σ_T). The adjusted odds ratios with 95% confidence intervals (black dots with error bars) for different lags are displayed.

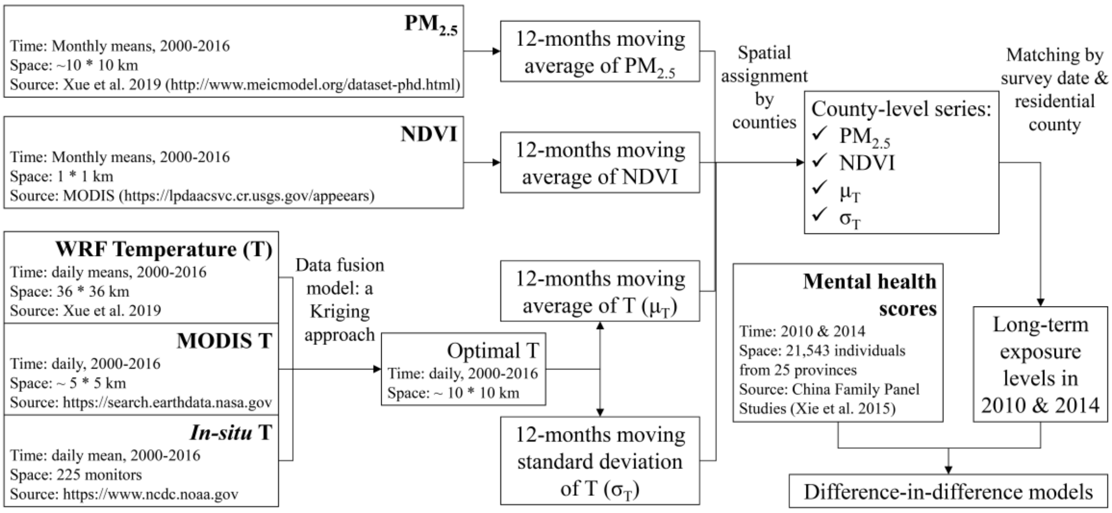

Supplementary Figure 4 Diagram of data preparation and analysis. The boxes highlighted by bolded titles present the directly inputted datasets into this study.

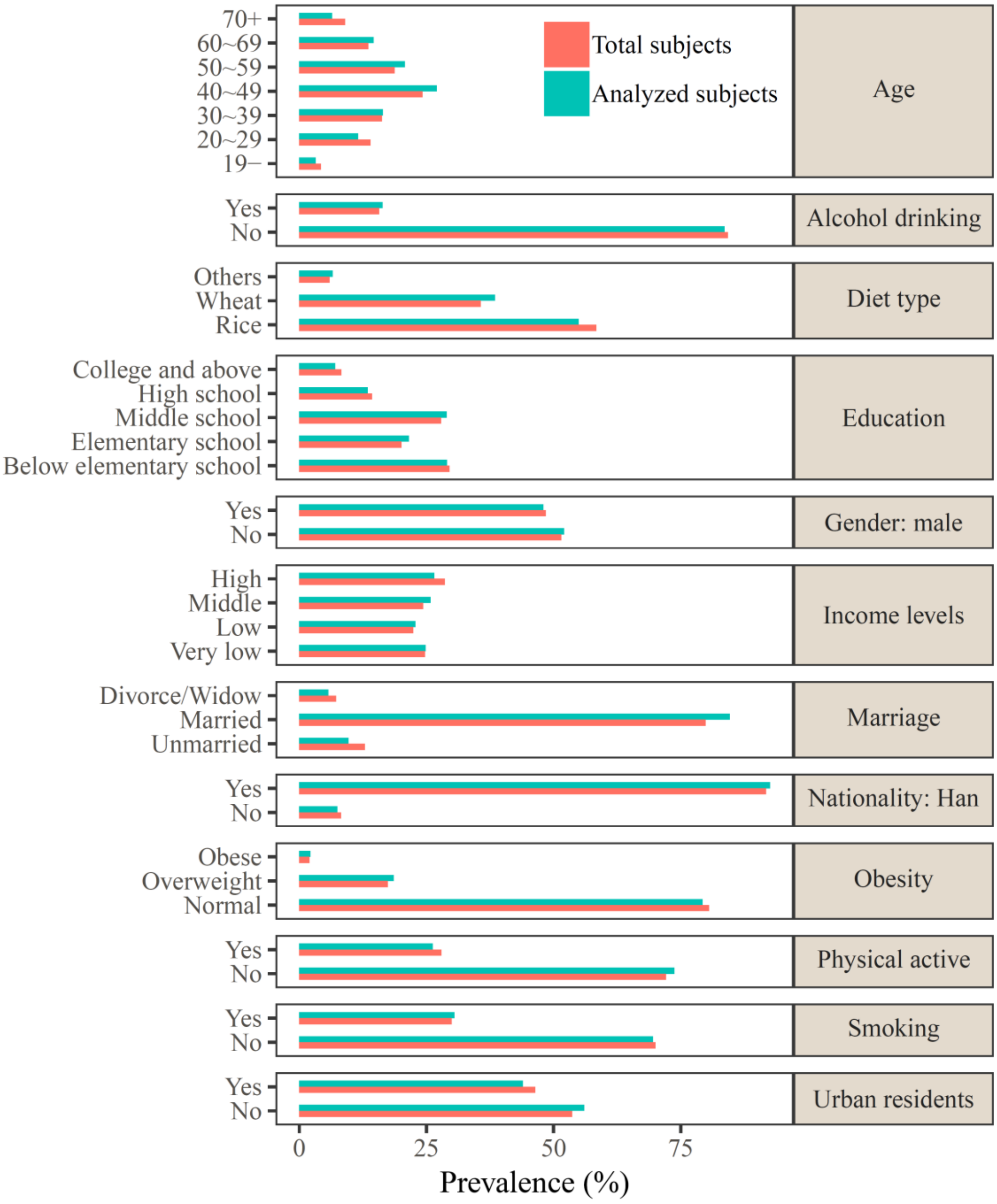

Supplementary Figure 5 A comparison between population characteristics of the total subjects surveyed by 2010 CFPS (red bars) and those of the analyzed subjects in this study (blue bars).

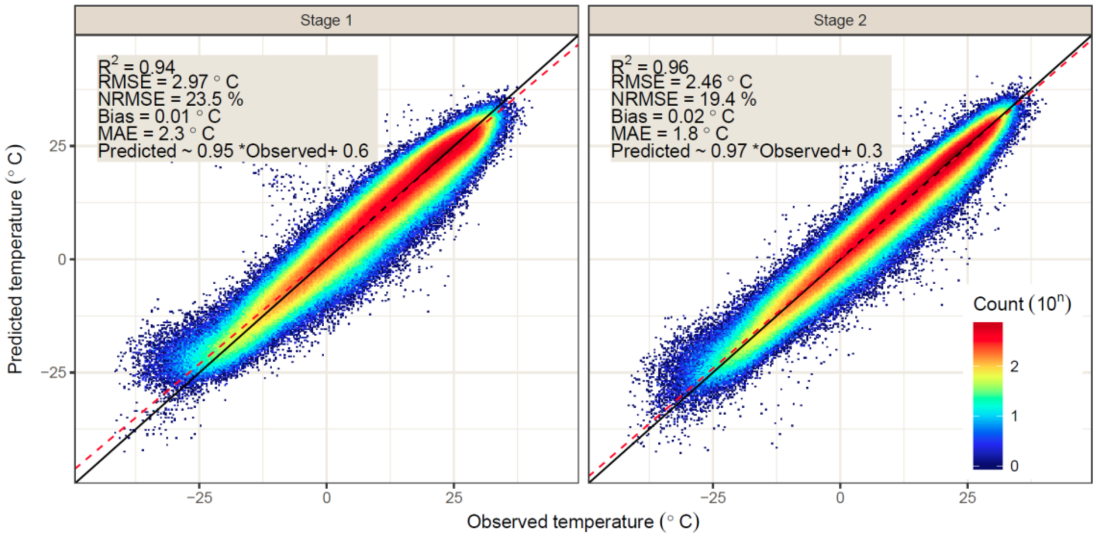

Supplementary Figure 6 Cross-validation for the fused estimates of temperature (2000-2016) in China. The color presents the density of the plotted samples (dots).

Supplementary Tables

Supplementary Table 1 Summary statistics on population characteristics and environmental exposure

Variable	Group	Total population	Subgroups by change in score		
			Decreased	Unchanged	Increased
Total population		Number (prevalence)			
		21,543 (100%)	8,729 (100%)	4,961 (100%)	7,853 (100%)
2010 baseline population characteristics		Number (prevalence)			
Age (years)	< 20	683 (3.2%)	246 (2.8%)	140 (2.8%)	297 (3.8%)
	20–29	2,494 (11.6%)	1,055 (12.1%)	492 (9.9%)	947 (12.1%)
	30–39	3,541 (16.4%)	1,463 (16.8%)	800 (16.1%)	1,278 (16.3%)
	40–49	5,815 (27.0%)	2,360 (27.0%)	1,302 (26.2%)	2,153 (27.4%)
	50–59	4,473 (20.8%)	1,780 (20.4%)	1,090 (22.0%)	1,603 (20.4%)
	60–69	3,147 (14.6%)	1,246 (14.3%)	797 (16.1%)	1,104 (14.1%)
	70+	1,390 (6.5%)	579 (6.6%)	340 (6.9%)	471 (6.0%)
Alcohol consumption	No	18,017 (83.6%)	7,314 (83.8%)	4,016 (81.0%)	6,687 (85.2%)
	Yes	3,525 (16.4%)	1,414 (16.2%)	945 (19.0%)	1,166 (14.8%)
	Unknown	1 (0.0%)	1 (0.0%)	0 (0.0%)	0 (0.0%)

Education	Below elementary school	6,249 (29.0%)	2,633 (30.2%)	1,135 (22.9%)	2,481 (31.6%)
	Elementary school	4,635 (21.5%)	1,835 (21.0%)	1,115 (22.5%)	1,685 (21.5%)
	Middle school	6,244 (29.0%)	2,554 (29.3%)	1,611 (32.5%)	2,079 (26.5%)
	High school	2,893 (13.4%)	1,103 (12.6%)	717 (14.5%)	1,073 (13.7%)
	College and above	1,517 (7.0%)	604 (6.9%)	380 (7.7%)	533 (6.8%)
	Unknown	5 (0.0%)	0 (0.0%)	3 (0.1%)	2 (0.0%)
Diet type (staple food)	Rice	11,835 (54.9%)	4,804 (55.0%)	2,903 (58.5%)	4,128 (52.6%)
	Wheat	8,290 (38.5%)	3,300 (37.8%)	1,779 (35.9%)	3,211 (40.9%)
	Other	1,417 (6.6%)	624 (7.1%)	279 (5.6%)	514 (6.5%)
	Unknown	1 (0.0%)	1 (0.0%)	0 (0.0%)	0 (0.0%)
Gender	Female	11,208 (52.0%)	4,740 (54.3%)	2,332 (47.0%)	4,136 (52.7%)
	Male	10,335 (48.0%)	3,989 (45.7%)	2,629 (53.0%)	3,717 (47.3%)
Income	Very low	5,032 (23.4%)	2,089 (23.9%)	1,170 (23.6%)	1,773 (22.6%)
	Low	4,759 (22.1%)	2,013 (23.1%)	877 (17.7%)	1,869 (23.8%)
	Middle	5,574 (25.9%)	2,184 (25.0%)	1,512 (30.5%)	1,878 (23.9%)

	High	5,398 (25.1%)	2,165 (24.8%)	1,249 (25.2%)	1,984 (25.3%)
	Unknown	780 (3.6%)	278 (3.2%)	153 (3.1%)	349 (4.4%)
Marriage	Married	18,230 (84.6%)	7,477 (85.7%)	4,273 (86.1%)	6,480 (82.5%)
	Single	2,081 (9.7%)	781 (8.9%)	434 (8.7%)	866 (11.0%)
	Divorced/widowed	1,226 (5.7%)	470 (5.4%)	254 (5.1%)	502 (6.4%)
	Unknown	6 (0.0%)	1 (0.0%)	0 (0.0%)	5 (0.1%)
Nationality	Han	19,895 (92.4%)	7,930 (90.8%)	4,640 (93.5%)	7,325 (93.3%)
	Non-Han	1,602 (7.4%)	780 (8.9%)	309 (6.2%)	513 (6.5%)
	Unknown	46 (0.2%)	19 (0.2%)	12 (0.2%)	15 (0.2%)
Physical activity	No	15,883 (73.7%)	6,554 (75.1%)	3,436 (69.3%)	5,893 (75.0%)
	Yes	5,648 (26.2%)	2,172 (24.9%)	1,521 (30.7%)	1,955 (24.9%)
	Unknown	12 (0.1%)	3 (0.0%)	4 (0.1%)	5 (0.1%)
Obesity status	Normal (BMI≤25 kg m^{-2})	16,557 (76.9%)	6,732 (77.1%)	3,699 (74.6%)	6,126 (78.0%)
	Overweight (BMI: 25~30 kg m^{-2})	3,911 (18.2%)	1,561 (17.9%)	1,034 (20.8%)	1,316 (16.8%)
	Obese (BMI > 30 kg m^{-2})	448 (2.1%)	179 (2.1%)	119 (2.4%)	150 (1.9%)
	Unknown	627 (2.9%)	257 (2.9%)	109 (2.2%)	261 (3.3%)
Residence	Rural	12,069 (56.0%)	4,936 (56.5%)	2,460 (49.6%)	4,673 (59.5%)

	Urban	9,474 (44.0%)	3,793 (43.5%)	2,501 (50.4%)	3,180 (40.5%)
Smoking	No	14,979 (69.5%)	6,176 (70.8%)	3,359 (67.7%)	5,444 (69.3%)
	Yes	6,564 (30.5%)	2,553 (29.2%)	1,602 (32.3%)	2,409 (30.7%)
Changes in population characteristics from 2010 to 2014		Number (prevalence)			
Alcohol consumption	From Yes to No	1,443 (6.7%)	614 (7.0%)	343 (6.9%)	486 (6.2%)
	Unchanged	18,666 (86.6%)	7,542 (86.4%)	4,271 (86.1%)	6,853 (87.3%)
	From No to Yes	1,432 (6.6%)	572 (6.6%)	346 (7.0%)	514 (6.5%)
	Unknown	2 (0.0%)	1 (0.0%)	1 (0.0%)	0 (0.0%)
Education	Unchanged	19,039 (88.4%)	7,707 (88.3%)	4,412 (88.9%)	6,920 (88.1%)
	Increased	2,499 (11.6%)	1,022 (11.7%)	546 (11.0%)	931 (11.9%)
	Unknown	5 (0.0%)	0 (0.0%)	3 (0.1%)	2 (0.0%)
Migration	No	18,538 (86.1%)	7,550 (86.5%)	4,223 (85.1%)	6,765 (86.1%)
	Yes	3,005 (13.9%)	1,179 (13.5%)	738 (14.9%)	1,088 (13.9%)
Obesity	Decreased	1,214 (5.6%)	519 (5.9%)	288 (5.8%)	407 (5.2%)
	Unchanged	16,921 (78.5%)	6,855 (78.5%)	3,917 (79.0%)	6,149 (78.3%)

	Increased	2,229 (10.3%)	865 (9.9%)	546 (11.0%)	818 (10.4%)
	Unknown	1,179 (5.5%)	490 (5.6%)	210 (4.2%)	479 (6.1%)
Physical activity	Decreased	2,197 (10.2%)	859 (9.8%)	531 (10.7%)	807 (10.3%)
	Unchanged	15,020 (69.7%)	6,119 (70.1%)	3,415 (68.8%)	5,486 (69.9%)
	Increased	4,297 (19.9%)	1,744 (20.0%)	1,010 (20.4%)	1,543 (19.6%)
	Unknown	29 (0.1%)	7 (0.1%)	5 (0.1%)	17 (0.2%)
Smoking	From Yes to No	1,167 (5.4%)	482 (5.5%)	273 (5.5%)	412 (5.2%)
	Unchanged	19,537 (90.7%)	7,896 (90.5%)	4,495 (90.6%)	7,146 (91.0%)
	From No to Yes	839 (3.9%)	351 (4.0%)	193 (3.9%)	295 (3.8%)
Changes in question-specific score from 2010 to 2014		Number (prevalence)			
Q_1	Decreased	6,265 (29.1%)	5,466 (62.6%)	347 (7.0%)	452 (5.8%)
	Unchanged	10,082 (46.8%)	2,834 (32.5%)	4,310 (86.9%)	2,938 (37.4%)
	Increased	5,196 (24.1%)	429 (4.9%)	304 (6.1%)	4,463 (56.8%)
Q_2	Decreased	5,701 (26.5%)	4,960 (56.8%)	306 (6.2%)	435 (5.5%)
	Unchanged	11,228 (52.1%)	3,450 (39.5%)	4,407 (88.8%)	3,371 (42.9%)

	Increased	4,614 (21.4%)	319 (3.7%)	248 (5.0%)	4,047 (51.5%)
Q_3	Decreased	5,220 (24.2%)	4,606 (52.8%)	251 (5.1%)	363 (4.6%)
	Unchanged	11,943 (55.4%)	3,826 (43.8%)	4,468 (90.1%)	3,649 (46.5%)
	Increased	4,380 (20.3%)	297 (3.4%)	242 (4.9%)	3,841 (48.9%)
Q_4	Decreased	3,524 (16.4%)	3,091 (35.4%)	160 (3.2%)	273 (3.5%)
	Unchanged	14,373 (66.7%)	5,296 (60.7%)	4,584 (92.4%)	4,493 (57.2%)
	Increased	3,646 (16.9%)	342 (3.9%)	217 (4.4%)	3,087 (39.3%)
Q_5	Decreased	5,127 (23.8%)	4,406 (50.5%)	289 (5.8%)	432 (5.5%)
	Unchanged	11,443 (53.1%)	3,826 (43.8%)	4,361 (87.9%)	3,256 (41.5%)
	Increased	4,973 (23.1%)	497 (5.7%)	311 (6.3%)	4,165 (53.0%)
Q_6	Decreased	3,400 (15.8%)	2,966 (34.0%)	153 (3.1%)	281 (3.6%)
	Unchanged	14,849 (68.9%)	5,423 (62.1%)	4,618 (93.1%)	4,808 (61.2%)
	Increased	3,294 (15.3%)	340 (3.9%)	190 (3.8%)	2,764 (35.2%)
Changes in environmental variables from 2010 to 2014		Mean (interquartile range)			

$PM_{2.5}$ ($\mu g\ m^{-3}$)	-0.66 (-3.56, 2.24)	-0.55 (-3.52, 2.20)	-0.85 (-3.67, 2.17)	-0.65 (-3.52, 2.28)
Normalized difference vegetation index (NDVI)	0.03 (0.01, 0.04)	0.02 (0.01, 0.04)	0.03 (0.01, 0.04)	0.03 (0.01, 0.04)
Mean temperature (μ_T, °C)	0.98 (0.22, 1.72)	0.97 (0.19, 1.72)	1.00 (0.23, 1.77)	0.99 (0.25, 1.69)
Temperature variance (σ_T, °C)	-0.55 (-1.04, -0.03)	-0.53 (-1.04, -0.01)	-0.57 (-1.01, -0.08)	-0.55 (-1.04, -0.03)

BMI, body mass index; $PM_{2.5}$, fine particulate matter

Supplementary Table 2 Pairwise Spearman correlation coefficients of the relationships between changes in mental health and environmental exposure.

	Q_1	Q_2	Q_3	Q_4	Q_5	Q_6	Score	$PM_{2.5}$	NDVI	μ_T
Q_2	0.48***									
Q_3	0.44***	0.53***								
Q_4	0.33***	0.34***	0.39***							
Q_5	0.35***	0.37***	0.39***	0.40***						
Q_6	0.31***	0.32***	0.35***	0.51***	0.42***					
Score	0.71***	0.71***	0.71***	0.61***	0.67***	0.59***				
$PM_{2.5}$	-0.01	-0.01*	-0.01	-0.01	0.00	0.00	-0.01			
NDVI	0.01	0.02***	0.02**	0.01	0.00	0.00	0.01	-0.21***		
μ_T	0.01	0.01	0.01	0.00	0.01*	0.00	0.02*	0.19***	0.20***	
σ_T	-0.01	-0.01	-0.01#	0.00	-0.01#	0.00	-0.02*	-0.12***	-0.24***	-0.78***

*** P-value < 0.001; ** P-value ~ (0.001, 0.01); * P-value ~ (0.01, 0.05); # P-value ~ (0.05, 0.1)

Question-specific and total scores were significantly positively correlated with each other. Total scores were most strongly correlated with Q_1, Q_2, or Q_3. Total scores were negatively correlated with $PM_{2.5}$ and σ_T, but positively correlated with NDVI and μ_T. The four environmental variables were significantly correlated with each other.

Supplementary Table 3 Estimated associations between total mental health scores and each of the four environmental factors.

Variable	Unit	Odds ratio per unit increment of variable (95% confidence intervals)				
		Model 1*	Model 2#	Model 3†	Model 4‡	Model 5※
$PM_{2.5}$	10 μg m^{-3}	0.89 (0.83, 0.96)	0.71 (0.61, 0.83)	0.71 (0.61, 0.83)	0.71 (0.61, 0.83)	0.72 (0.61, 0.84)
NDVI	0.05	1.05 (0.99, 1.11)	1.18 (1.08, 1.30)	1.19 (1.08, 1.30)	1.18 (1.08, 1.30)	1.19 (1.08, 1.30)
μ_T	1 °C	1.04 (1.01, 1.07)	0.98 (0.86, 1.12)	0.98 (0.85, 1.12)	0.97 (0.85, 1.11)	0.97 (0.85, 1.11)
σ_T	1 °C	0.94 (0.90, 0.98)	0.87 (0.77, 0.98)	0.87 (0.76, 0.98)	0.86 (0.76, 0.98)	0.85 (0.75, 0.97)

* Model 1: unadjusted model;

# Model 2: adjusted by spatial autocorrelation only;

† Model 3: adjusted for changes in individual-level variables, including alcohol consumption, education, migration, obesity, physical activity, and smoking status.

‡ Model 4: adjusted for baseline in individual-level variables, including age, alcohol consumption, education, dietary style, gender, income, marital status, nationality, physical activity, obesity, residence, and smoking status.

※Model 5: fully adjusted model.

$PM_{2.5}$, fine particulate matter; NDVI, normalized difference vegetation index; μ_T, mean temperature; σ_T, temperature variability

Supplementary Table 4 The associations between mental health score (MHS) and environmental variables, estimated by different types of models.

Variable	Unit	Odds ratio per unit increment of variable (95% confidence intervals)		Score change per unit increment of variable (95% confidence intervals)	
		Logit model*	Ordinal model#	Linear effect model†	Mixed effect model‡
$PM_{2.5}$	10 μg m^{-3}	0.72 (0.61, 0.84)	0.78 (0.69, 0.89)	-0.43 (-0.78, -0.08)	-0.72 (-1.44, -0.00)
NDVI	0.05	1.19 (1.08, 1.30)	1.10 (1.02, 1.19)	0.16 (-0.05, 0.37)	0.30 (-0.35, 0.95)
μ_T	1 °C	0.97 (0.85, 1.11)	0.98 (0.87, 1.09)	-0.10 (-0.40, 0.21)	-0.42 (-0.83, 0.00)
σ_T	1 °C	0.85 (0.75, 0.97)	0.87 (0.78, 0.96)	-0.44 (-0.72, -0.15)	-0.60 (-0.95, -0.24)

* Logit model: the model utilized in the main-text (Equation 1).

Ordinal model:

In the ordinal model, the dependent variable (y) is set as the ordinal change in total MHS ($\Delta\Sigma_i Q_i \in$ [-24, -23, …, 23, 24]). The independent variables (x and z) are set as the same as the logit model (Equation 1). For instance, x is set as the change in one of the four environmental variables (*i.e.*, $\Delta PM_{2.5}$, ΔNDVI, $\Delta\mu_T$ and $\Delta\sigma_T$). The regression coefficient (β_x) for an environmental variable, *x,* can be interpreted as a logarithmic scale of odds ratios (ORs) for increments in the score after per-unit increments in *x*. Specifically, for a given level of environmental exposure (x), the OR for an increment of a given score (y) can be denoted in terms of ORs (y | x) = P(score > y | x) / P(score ≤ y | x), where P(•) indicates the cumulative probability function; after an increment in the environmental variable ($x + \Delta x$), the ratio of odds (y | $x + \Delta x$) against odds (y | x) can be calculated as exp ($\Delta x\beta_x$). As the OR of exp ($\Delta x\beta_x$) is independent of the given level of the score (y), the ordinal logistic model is also known as the proportional odds model. The no-effect reference is set as OR = 1.

† Linear effect model:

In the linear effect model, the dependent variable (y) is set as the continuous change in total MHS ($\Delta\Sigma_i Q_i \in$ [-24, 24]). The independent variables (x and z) are set as the same as the logit model (Equation 1). The regression coefficient (β_x) for an environmental variable (x) is directly interpreted as its effect on mental health. In other words, the effect is quantified as score change for per-unit change in the environmental variable. The no-effect reference is set as $\beta_x = 0$.

‡ Mixed effect model:

In the linear mixed effect model, the dependent variable (y) is set as the continuous variable of total MHS ($\sum_i Q_i \in [1, 25]$). The independent variables (x and z) are set as one of the four environmental factors (*i.e.*, $PM_{2.5}$, NDVI, μ_T or σ_T) and the individual-level characteristics in 2010 or 2014. An extra term of the random intercept is incorporated to control the person-specific effect on mental health. Effect interpretation is identical to that of the linear effect model (*e.g.*, no-effect reference: $\beta_x = 0$).

Supplementary Software

1. *Codes for the regression models in the main-text*

```
########################################################
#R codes for the main models used in the following manuscript
#Declines in mental health associated with air pollution and temperature variability in China
#Authors: Tao Xue, Tong Zhu, Yixuan Zheng, Qiang Zhang
#The code is not utalized for runing, but to aid interpretation of the statistical models used in the study.
#For more information, questions, or data requirements, please email Dr. Tao Xue (xuetaogk@126.com).

#load the package used in statistical inference
library(mgcv)
#load the analyzed data
load("AGT.RData")

#construct the logit models used in the main-text (Table S3 & Figure 2)
for(y in c("score",paste("Q",1:6,sep="")))
  for(x in c("PM25","NDVI","TMP.mu","TMP.sd"))
  {
    f<-paste(y,"~",x,sep="")
    model1<-gam(as.formula(f),data = AGT,family=binomial)
    tmp0<-as.data.frame(summary(model1)$p.table)[x,]

    f<-paste(y,"~",x,"+s(lon, lat, bs = 'sos')",sep="")
    model2<-gam(as.formula(f),data = AGT,family=binomial)
    tmp1<-as.data.frame(summary(model2)$p.table)[x,]

    f<-paste(y,"~",x,"+s(lon, lat, bs = 'sos') + mig + edu_inc + BMI_chg + phyactive_chg + smoke_chg + drink_chg",sep="")
    model3<-gam(as.formula(f),data = AGT,family=binomial)
    tmp2<-as.data.frame(summary(model3)$p.table)[x,]

    f<-paste(y,"~",x,"+s(lon, lat, bs = 'sos') + urban + age + race + gender + education + income + marry + BMI + smoke + drink + phyactive + food",sep="")
    model4<-gam(as.formula(f),data = AGT,family=binomial)
    tmp3<-as.data.frame(summary(model4)$p.table)[x,]

    f<-paste(y,"~",x,"+s(lon, lat, bs = 'sos') + mig + edu_inc + BMI_chg + phyactive_chg + smoke_chg + drink_chg + urban + age + race + gender + education + income + marry + BMI + smoke + drink + phyactive + food",sep="")
    model5<-gam(as.formula(f),data = AGT,family=binomial)
    tmp4<-as.data.frame(summary(model5)$p.table)[x,]
```

```
    tmp=cbind(model=0:4,x=x,y=y,rbind(tmp0,tmp1,tmp2,tmp3,tmp4))
    if(y=="score"&x=="PM25") TabS3=tmp else TabS3<-rbind(TabS3,tmp)
  }

#construct the nonlinear models used in the main-text (Figure 1)
nonlinear.models<-list(PM25=NA,
              NDVI=NA,
              TMP.mu=NA,
              TMP.sd=NA)
for(y in c("score"))
  for(x in c("PM25","NDVI","TMP.mu","TMP.sd"))
  {
    f<-paste(y,"~s(",x,",bs='cr',fx=F)+s(lon, lat, bs = 'sos') + mig + edu_inc + BMI_chg +
phyactive_chg + smoke_chg + drink_chg + urban + age + race + gender + education +
income + marry + BMI + smoke + drink + phyactive + food",sep="")
    nonlinear.models[[x]]<-gam(as.formula(f), data = AGT,family = binomial)
  }

#construct the double-exposure models used in the main-text (Figure S)
n2<-combn(c("PM25","NDVI","TMP.mu","TMP.sd"),2)
n2=apply(n2,2,paste,collapse="+")
for(y in c("score"))
  for(x in n2)
  {
    f<-paste(y,"~",x,"+s(lon, lat, bs = 'sos') + mig + edu_inc + BMI_chg + phyactive_chg +
smoke_chg + drink_chg + urban + age + race + gender + education + income + marry +
BMI + smoke + drink + phyactive + food",sep="")
    m<-gam(as.formula(f),data = AGT,family = binomial)
    tmp=as.data.frame(summary(m)$p.table)
    id<-which(rownames(tmp)%in%c("PM25","NDVI","TMP.mu","TMP.sd"))
    tmp=tmp[id,]
    tmp=cbind(model=3,x=x,y=y,x2=rownames(tmp),tmp)
    if(y=="score"&x==n2[1]) TwoExpo.models=tmp else TwoExpo.models<-
rbind(TwoExpo.models,tmp)
  }
```

2. *Codes for Figure 1*

```
####################################################################
#R codes to generate Figure 1 in the following manuscript
#Declines in mental health associated with air pollution and temperature variability in China
#Authors: Tao Xue, Tong Zhu, Yixuan Zheng, Qiang Zhang
#For more information, questions, or data requirements, please email Dr. Tao Xue
(xuetaogk@126.com).
library(ggplot2)
```

```
library(gridExtra)
load("3a Data of figure 1.RData")

Fig1<-ggplot(data=cbind(rbind(subset(data,x=="PM25"),
                              subset(data,x=="PM25")[1,]),ref=1))+
  geom_path(aes(x=y2,y=ref),linetype=1,col="grey")+
  geom_path(aes(x=y2,y=lo),linetype=2)+
  geom_path(aes(x=y2,y=up),linetype=2)+
  geom_path(aes(x=y2,y=mid))+
  geom_point(aes(x=y2,y=mid))+
  coord_radar()+
  theme_bw()+
scale_x_continuous(limits=c(1,7),breaks=c(1:6),labels=parse(text=paste("italic(Q)[",1:6,"]",s
ep="")))+
  theme(axis.title.x = element_blank(),panel.grid.minor.x=element_blank(),
        panel.grid.major.y=element_blank(),
        text=element_text(family='Times'))+
  ylab(expression("Odds Ratio per"~10~mu~g~m^'-3'~"increment of"~PM[2.5]))

Fig2<-ggplot(data=cbind(rbind(subset(data,x=="NDVI"),
                              subset(data,x=="NDVI")[1,]),ref=1))+
  geom_path(aes(x=y2,y=ref),linetype=1,col="grey")+
  geom_path(aes(x=y2,y=lo),linetype=2)+
  geom_path(aes(x=y2,y=up),linetype=2)+
  geom_path(aes(x=y2,y=mid))+
  geom_point(aes(x=y2,y=mid))+
  coord_radar()+
  theme_bw()+
scale_x_continuous(limits=c(1,7),breaks=c(1:6),labels=parse(text=paste("italic(Q)[",1:6,"]",s
ep="")))+
  theme(axis.title.x = element_blank(),panel.grid.minor.x=element_blank(),
        panel.grid.major.y=element_blank(),
        text=element_text(family='Times'))+
  ylab(expression("Odds Ratio per"~0.05~"increment of"~NDVI))

Fig3<-ggplot(data=cbind(rbind(subset(data,x=="TMP.mu"),
                              subset(data,x=="TMP.mu")[1,]),ref=1))+
  geom_path(aes(x=y2,y=ref),linetype=1,col="grey")+
  geom_path(aes(x=y2,y=lo),linetype=2)+
  geom_path(aes(x=y2,y=up),linetype=2)+
  geom_path(aes(x=y2,y=mid))+
  geom_point(aes(x=y2,y=mid))+
  coord_radar()+
  theme_bw()+
```

```
scale_x_continuous(limits=c(1,7),breaks=c(1:6),labels=parse(text=paste("italic(Q)[",1:6,"]",sep="")))+
  theme(axis.title.x = element_blank(),panel.grid.minor.x=element_blank(),
        panel.grid.major.y=element_blank(),
        text=element_text(family='Times'))+
  ylab(expression("Odds Ratio per"~1~degree~C~"increment of"~mu[T]))

Fig4<-ggplot(data=cbind(rbind(subset(data,x=="TMP.sd"),
                              subset(data,x=="TMP.sd")[1,]),ref=1))+
  geom_path(aes(x=y2,y=ref),linetype=1,col="grey")+
  geom_path(aes(x=y2,y=lo),linetype=2)+
  geom_path(aes(x=y2,y=up),linetype=2)+
  geom_path(aes(x=y2,y=mid))+
  geom_point(aes(x=y2,y=mid))+
  coord_radar()+
  theme_bw()+
scale_x_continuous(limits=c(1,7),breaks=c(1:6),labels=parse(text=paste("italic(Q)[",1:6,"]",sep="")))+
  theme(axis.title.x = element_blank(),panel.grid.minor.x=element_blank(),
        panel.grid.major.y=element_blank(),
        text=element_text(family='Times'))+
  ylab(expression("Odds Ratio per"~1~degree~C~"increment of"~sigma[T]))

pdf("Figure 1.pdf",height=7,width=7.2)
grid.arrange(Fig1,Fig2,Fig3,Fig4)
dev.off()
```

3. *Codes for Figure 2*

```
##################################################################
#R codes to generate Figure 2 in the following manuscript
#Declines in mental health associated with air pollution and temperature variability in China
#Authors: Tao Xue, Tong Zhu, Yixuan Zheng, Qiang Zhang
#For more information, questions, or data requirements, please email Dr. Tao Xue (xuetaogk@126.com).

library(ggplot2)
library(gridExtra)
load("3b Data of figure 2.RData")
Fig1<-ggplot(data=data1$prd)+
  geom_hline(aes(yintercept=1-data1$lo0),linetype=1,color="grey50")+
  geom_col(data=data1$frq,aes(x=x,y=den2),fill="grey70",alpha=0.5)+
  geom_path(aes(x=x,y=lo-data1$lo0),linetype=2)+
  geom_path(aes(x=x,y=up-data1$lo0),linetype=2)+
  geom_path(aes(x=x,y=fit-data1$lo0))+
  theme_classic()+
```

```
  xlab(expression(PM[2.5]~change~(mu~g~m^'-3')))+
  ylab("Odds ratio")+
  scale_y_continuous(breaks = seq(0,2,0.2)-data1$lo0,labels = seq(0,2,0.2),
                     sec.axis = dup_axis(~.,breaks=seq(0,20,2) * data1$scl, labels = seq(0,20,2), name = expression("Probability density [% per (2" ~ mu ~ g~m^'-3' ~ ")]")))+
  theme(axis.text.y.right = element_text(color="grey70"),
        axis.title.y.right = element_text(color="grey70"),
        text=element_text(family='Times'))

Fig2<-ggplot(data=data2$prd)+
  geom_hline(aes(yintercept=1-data2$lo0),linetype=1,color="grey50")+
  geom_col(data=data2$frq,aes(x=x/10000,y=den2),fill="grey70",alpha=0.5)+
  geom_path(aes(x=x/10000,y=lo-data2$lo0),linetype=2)+
  geom_path(aes(x=x/10000,y=up-data2$lo0),linetype=2)+
  geom_path(aes(x=x/10000,y=fit-data2$lo0))+
  theme_classic()+
  xlab(expression("NDVI change"))+
  ylab("Odds ratio")+
  scale_y_continuous(breaks = seq(0,2,0.1)-data2$lo0,labels = seq(0,2,0.1),
                     sec.axis = dup_axis(~.,breaks = seq(0,20,2) * data2$scl,labels = seq(0,20,2), name = expression("Probability density (% per " ~ "0.01" ~ ")")))+
  theme(axis.text.y.right = element_text(color="grey70"),
        axis.title.y.right = element_text(color="grey70"),
        text=element_text(family='Times'))

Fig3<-ggplot(data=data3$prd)+
  geom_hline(aes(yintercept=1-data3$lo0),linetype=1,color="grey50")+
  geom_col(data=data3$frq,aes(x=x,y=den2),fill="grey70",alpha=0.5)+
  geom_path(aes(x=x,y=lo-data3$lo0),linetype=2)+
  geom_path(aes(x=x,y=up-data3$lo0),linetype=2)+
  geom_path(aes(x=x,y=fit-data3$lo0))+
  theme_classic()+
  xlab(expression(mu[T]~change~(degree~C)))+
  ylab("Odds ratio")+
  scale_y_continuous(breaks=seq(0,2,0.1)-data3$lo0,labels=seq(0,2,0.1),
                     sec.axis = dup_axis(~.,breaks = seq(0,10,1) * data3$scl,labels = seq(0,10,1),name = expression("Probability density [% per (0.2" ~ degree~C ~ ")]")))+
  theme(axis.text.y.right = element_text(color="grey70"),
        axis.title.y.right = element_text(color="grey70"),
        text=element_text(family='Times'))

Fig4<-ggplot(data=data4$prd)+
  geom_hline(aes(yintercept=1-data4$lo0),linetype=1,color="grey50")+
  geom_col(data=data4$frq,aes(x=x,y=den2),fill="grey70",alpha=0.5)+
```

```
  geom_path(aes(x=x,y=lo-data4$lo0),linetype=2)+
  geom_path(aes(x=x,y=up-data4$lo0),linetype=2)+
  geom_path(aes(x=x,y=fit-data4$lo0))+
  theme_classic()+
  xlab(expression(sigma[T]~change~(degree~C)))+
  ylab("Odds ratio")+
  scale_y_continuous(breaks=seq(0,2,0.1)-data4$lo0,labels=seq(0,2,0.1),
                     sec.axis = dup_axis(~.,breaks = seq(0,10,1) * data4$scl,labels = 
seq(0,10,1),name = expression("Probability density [% per (0.2"~degree~C~")]")))+
  theme(axis.text.y.right = element_text(color="grey70"),
        axis.title.y.right = element_text(color="grey70"),
        text=element_text(family='Times'))

pdf("Figrue 2.pdf",height=6,width=8)
grid.arrange(Fig1,Fig2,Fig3,Fig4,ncol=2)
dev.off()
```

4. *Codes for Figure 3*

```
##################################################################
#R codes to generate Figure 3 in the following manuscript
#Declines in mental health associated with air pollution and temperature variability in China
#Authors: Tao Xue, Tong Zhu, Yixuan Zheng, Qiang Zhang
#For more information, questions, or data requirements, please email Dr. Tao Xue 
(xuetaogk@126.com).

library(ggplot2)
load("3c Data of figure 3.RData")
Fig<-ggplot(data=data)+
  geom_hline(aes(yintercept=1),linetype=2,color="grey")+
  geom_linerange(aes(x=lab,ymin=lo,ymax=up))+
  geom_point(aes(x=lab,y=mid),shape=rep(c(21,16,16,16),4),fill="white")+
  facet_wrap(~x2,scales="free_y",ncol=1,strip.position = "bottom",
             labeller = label_parsed
  )+
  coord_flip()+
  theme_classic()+
  theme(axis.title = element_blank(),text=element_text(family = "Times"))+
  scale_x_discrete(labels=labs)

pdf("Figure 3 demo.pdf",height=5,width=5)
print(Fig)
dev.off()
```
