# Supplementary material for: Declines in mental health associated with air pollution and temperature variability in China
Source: Nat Commun. 2019 May 15;10:2165. doi: 10.1038/s41467-019-10196-y (PMC6520357; doi:10.1038/s41467-019-10196-y)
Supplement: Supplementary file 2 — Reporting Summary [file 41467_2019_10196_MOESM2_ESM.pdf]

nature research

Corresponding author(s): Tong Zhu

Last updated by author(s): Apr 11, 2019

Reporting Summary

Nature Research wishes to improve the reproducibility of the work that we publish. This form provides structure for consistency and transparency in reporting. For further information on Nature Research policies, see Authors & Referees and the Editorial Policy Checklist.

Statistics

For all statistical analyses, confirm that the following items are present in the figure legend, table legend, main text, or Methods section.

n/a	Confirmed	
☐	☒	The exact sample size (*n*) for each experimental group/condition, given as a discrete number and unit of measurement
☐	☒	A statement on whether measurements were taken from distinct samples or whether the same sample was measured repeatedly
☐	☒	The statistical test(s) used AND whether they are one- or two-sided<br>*Only common tests should be described solely by name; describe more complex techniques in the Methods section.*
☐	☒	A description of all covariates tested
☐	☒	A description of any assumptions or corrections, such as tests of normality and adjustment for multiple comparisons
☐	☒	A full description of the statistical parameters including central tendency (e.g. means) or other basic estimates (e.g. regression coefficient) AND variation (e.g. standard deviation) or associated estimates of uncertainty (e.g. confidence intervals)
☐	☒	For null hypothesis testing, the test statistic (e.g. *F*, *t*, *r*) with confidence intervals, effect sizes, degrees of freedom and *P* value noted<br>*Give P values as exact values whenever suitable.*
☒	☐	For Bayesian analysis, information on the choice of priors and Markov chain Monte Carlo settings
☒	☐	For hierarchical and complex designs, identification of the appropriate level for tests and full reporting of outcomes
☒	☐	Estimates of effect sizes (e.g. Cohen's *d*, Pearson's *r*), indicating how they were calculated

Our web collection on statistics for biologists contains articles on many of the points above.

Software and code

Policy information about availability of computer code

Data collection	All of the collected data including China Family Panel Studies (CFPS) can be directly downloaded from the websites listed in the data availability statement. All data were processed using in R.
Data analysis	All data analyses were using R (ver. 3.4.1), a publicly-available statistical software (https://www.r-project.org/) . The R codes are available from the corresponding author upon reasonable request.

For manuscripts utilizing custom algorithms or software that are central to the research but not yet described in published literature, software must be made available to editors/reviewers. We strongly encourage code deposition in a community repository (e.g. GitHub). See the Nature Research guidelines for submitting code & software for further information.

Data

Policy information about availability of data

All manuscripts must include a data availability statement. This statement should provide the following information, where applicable:

- Accession codes, unique identifiers, or web links for publicly available datasets
- A list of figures that have associated raw data
- A description of any restrictions on data availability

The population data (CFPS) that support the findings of this study are available from http://opendata.pku.edu.cn/. The NDVI data that support the findings of this study are available from https://lpdaacsvc.cr.usgs.gov/appeears/. The PM2.5 data that support the findings of this study are available from http://www.meicmodel.org/dataset-phd.html. The temperature data that support the findings of this study are available from https://www.ncdc.noaa.gov/ and https://search.earthdata.nasa.gov/.

Field-specific reporting

Please select the one below that is the best fit for your research. If you are not sure, read the appropriate sections before making your selection.

☐ Life sciences ☒ Behavioural & social sciences ☐ Ecological, evolutionary & environmental sciences

For a reference copy of the document with all sections, see nature.com/documents/nr-reporting-summary-flat.pdf

Behavioural & social sciences study design

All studies must disclose on these points even when the disclosure is negative.

Study description	Data are quantitative observational, derived from 2010 and 2014 surveys in China Family Panel Studies (CFPS).
Research sample	Research sample is 21,543 Chinese adults, with a sex ratio close to 1:1, as measured twice in 2010 and 2014 CFPS.
Sampling strategy	The CFPS used a multi-stage probability-based sampling design. At each stage, implicit stratification was employed to improve efficiency. Briefly, at the first stage, official administrative entities (counties/districts) were sampled, then communities (administrative villages/ resident committees) were selected from each administrative entity in the second stage. In the third stage, households were systematically sampled in each community.
Data collection	We rely on household surveys collected by CFPS, which followed the sampling protocol above. The CFPS surveyed > 30,000 adults from 25 provincial regions of China on multiple occasions from 2010. Data on personal characteristics (e.g., age), socioeconomic status (e.g., education and income), behavior patterns (e.g., physical activity), lifestyle (e.g., diet type), mental health status, and so on were collected by trained interviewers using standard questionnaires.
Timing	This study was based on two waves of CFPS in 2010 and 2014.
Data exclusions	In total, 25,618 of the 33,600 adults surveyed in 2010, and the 37,147 adults surveyed in 2014, participated in both evaluations. After excluding surveys with (1) incomplete answers to the mental health questionnaire or (2) a failure of geocoding, the data obtained from 21,543 adults from 25 provinces during the first and second surveys were included in the final analysis.
Non-participation	Detailed information on non-response rates were not available from CFPS. We use every observation reported in the 2010 and 2014 CFPS surveys.
Randomization	As described in the paper, the difference-in-difference design controlled the covariates (e.g., genetic factors) that do not change with time. The regression models additionally adjusted (1) the temporal changes in alcohol consumption, education, migration, obesity, physical activity, and smoking, and (2) 2010 baseline statues of age, alcohol consumption, education, diet type, gender, income level, marital status, nationality, physical activity status, obesity status, area of residence (urban or rural), and smoking status.

Reporting for specific materials, systems and methods

We require information from authors about some types of materials, experimental systems and methods used in many studies. Here, indicate whether each material, system or method listed is relevant to your study. If you are not sure if a list item applies to your research, read the appropriate section before selecting a response.

Materials & experimental systems

n/a	Involved in the study	
☒	☐	Antibodies
☒	☐	Eukaryotic cell lines
☒	☐	Palaeontology
☒	☐	Animals and other organisms
☒	☐	Human research participants
☒	☐	Clinical data

Methods

n/a	Involved in the study	
☒	☐	ChIP-seq
☒	☐	Flow cytometry
☒	☐	MRI-based neuroimaging
