# Supplementary material for: Declines in mental health associated with air pollution and temperature variability in China
Source: Nat Commun. 2019 May 15;10:2165. doi: 10.1038/s41467-019-10196-y (PMC6520357; doi:10.1038/s41467-019-10196-y)
Supplement: Supplementary file 3 — Description of Additional Supplementary Files [file 41467_2019_10196_MOESM3_ESM.pdf]

Description of Additional Supplementary Files

Supplemental Software:
Declines in mental health associated with air pollution and temperature variability in China

1 Main models: The demo R codes for the main models used in the manuscript.
2 (a-c): The R codes to generate the Figures in the manuscript.
3 (a-c): The R data to generate the Figures in the manuscript.
